# Solitary Fibrous Tumor: Case Report of Intrapulmonary Location

**DOI:** 10.1155/2018/5745471

**Published:** 2018-12-02

**Authors:** Adil Arsalane, Abdelfetah Zidane, Hicham Fenane, Amine Azami, Ismail Essadi, Abderrahim Raissi, Issam Lalya, Yacine Msougar

**Affiliations:** ^1^Department of Thoracic Surgery, Military Teaching Hospital Ibn Sina, University Cadi Ayyad, Morocco; ^2^Department of Thoracic Surgery, Teaching Hospital Mohammed VI, University Cadi Ayyad, Morocco; ^3^Department of Histopathology, Military Teaching Hospital Ibn Sina, University Cadi Ayyad, Morocco; ^4^Department of Medical Oncology, Military Teaching Hospital Ibn Sina, University Cadi Ayyad, Morocco

## Abstract

Solitary fibrous tumors are relatively rare neoplasms that commonly occur in the pleura, especially visceral pleura. However, an intrapulmonary site of this kind of tumors is even rarer. These tumors can be characterized by a heterogeneous evolution and have a benign or malignant behavior. Wide surgical resection is essential to cure the patient and to avoid recurrence. We present here the clinical, imaging, and histological features of a case with solitary fibrous tumor growing inside the lung.

## 1. Introduction

Solitary fibrous tumors (SFTs) are relatively rare neoplasms that commonly occur in the pleura, especially visceral leaflet of the pleura [1, 2]. They represent less than 5% of all pleural tumors [3]. Intrapulmonary SFTs are extremely rare. Some authors suggested that the origin of SFTs is mesenchymal cells derived from the submesothelial tissue of the pleura [4]. These tumors are characterized by a heterogeneous behavior. Most of the time they have benign evolution but sometimes they can take a malignant attitude [5–7]. Diagnosis is based on the histologic examination of the tumor after surgical resection which must be as wide as possible to avoid locoregional recurrence [1–7].

## 2. Case Presentation

**Figure 1:**
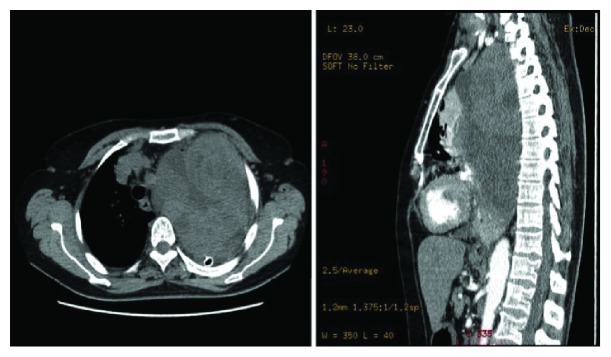
Chest computed tomography scan of an intrapulmonary solitary fibrous tumor, largely occupying the left hemithorax (mediastinal window).

**Figure 2:**
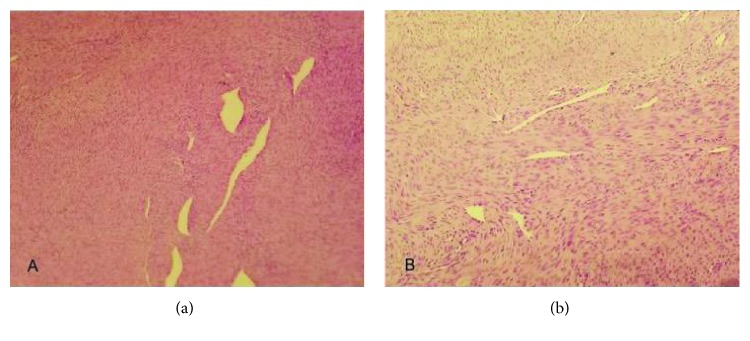
Proliferation of spindle or oval cells, arranged in a fascicular fashion with ropey collagen fibres, associated with variably dilated blood vessels often displaying staghorn-like appearance. Mitotic figures were few ((a) ×100, (b) ×200).

**Figure 3:**
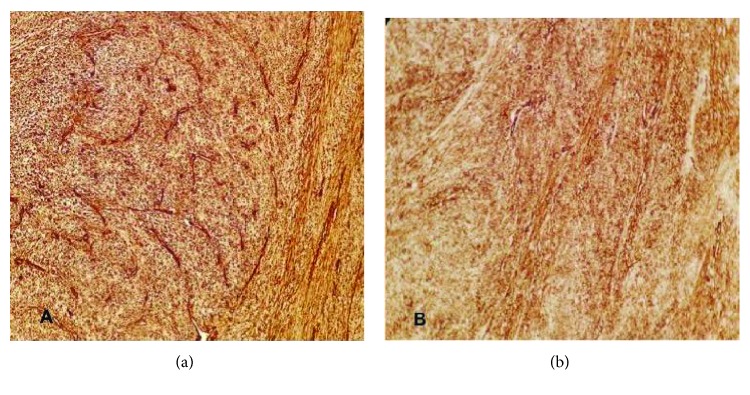
The spindle cells show strong and diffuse positivity for (a) CD34 and (b) bcl2; (×200).

**Figure 4:**
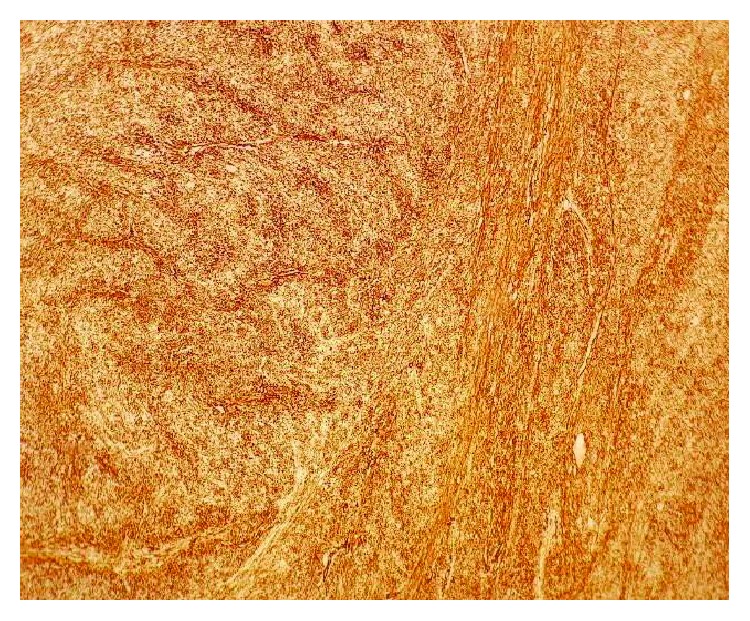
The spindle cells show positivity for vimentin (×200).

**Figure 5:**
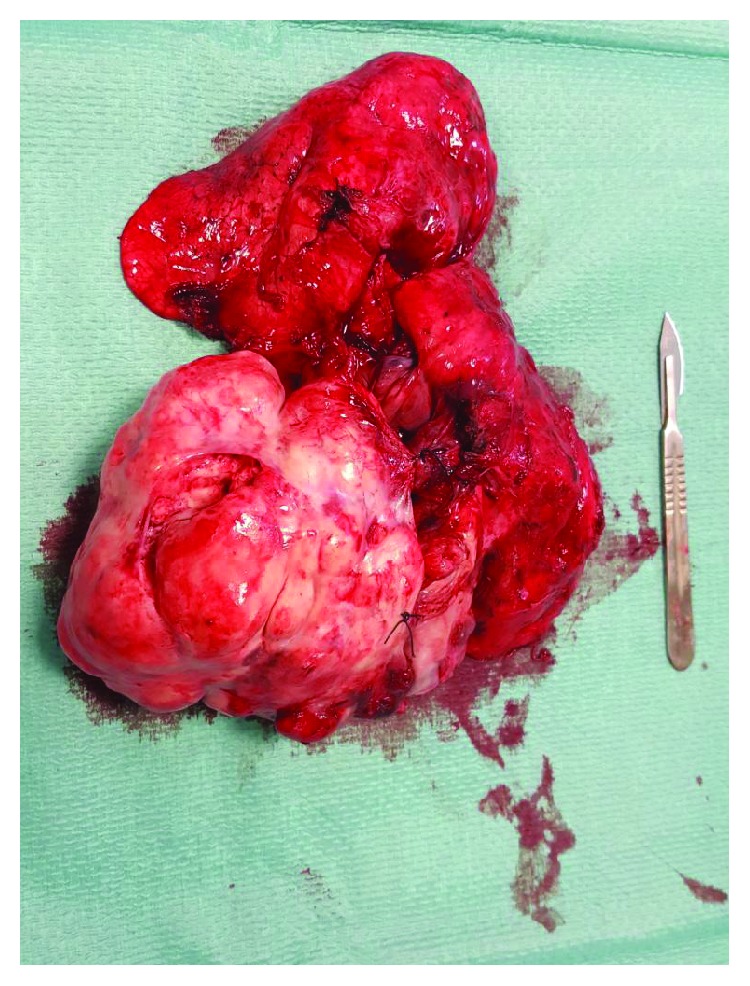
Pneumonectomy piece.

A 64-year-old Moroccan female was referred to our institution for chest pain, cough, dyspnea, and a large abnormal image at the left lung field on standard radio. She had no history of smoking or exposure to any chemical substances (asbestos). Chest physical examination has noted a left pleural effusion syndrome. Thoracocentesis was immediately performed, cleaned out 1000 ml of yellow fluid which was transudative. Routine blood tests were normal. A chest CT scan revealed a large necrotic and heterogeneous mass occupying almost all the left hemithorax (Figure 1). A transparietal biopsy of the mass was conducted and showed only fragmented fibrotic tissue. Therefore, a thoracoscopy exploration was performed showing a large pulmonary mass. A biopsy under thoracoscopy with histopathological study showed a proliferation of spindle cells with regions of hypercellularity admixed with hypocellular regions, accompanied by a collagenous stroma with branching hemangiopericytoma-like vessels. The neoplastic cells presented a low mitotic activity (2 mitoses per high-power field) without atypia or necrosis (Figure 2). Immunohistochemical staining was positive for CD34, bcl2 (Figure 3), and vimentin (Figure 4) but was negative for cytokeratin, SMA, desmin, and S100. The diagnosis of SFT was made. The excision of the mass was planned and a left posterolateral thoracotomy was realized. There was a hard mass invading the lower lobe of the lung with fissure encroaching and overrun of the proximal upper lobe parenchyma. Therefore, a pneumonectomy was performed (Figure 5). The suture line was covered with a pedicled pleural flap in order to prevent air leakage. The patient had a total postoperative recovery and was discharged on the 10th day after surgery. At 12-month follow-up, the patient was asymptomatic and a control CT scan showed no evidence of recurrence.

## 3. Discussion

Solitary fibrous tumor is an uncommon spindle cell tumor which arise mostly from the visceral pleura. With an incidence of less than 3 per 100000 hospital patients and less than 1000 cases described in the literature, it accounts for almost 5% of all pleural tumors [8]. Most of these tumors grow up into the pleural cavity with the presence of pedicle [2, 4, 9]. Its development inside the lung from the visceral pleura is uncommon [10]. Fewer than 20 cases have been reported in the literature [2]. It was thought to be from the submesothelial connective tissue [11]. These tumors which are unrelated to asbestos or smoking exposure are more frequently encountered between the fifth and eighth decades of life with no sex predilection [7, 10–14]. The clinical features depend on the site, size, and malignant potential of the tumor. These tumors are often found incidentally at standard chest X-ray. In our case, there were some symptoms such as chest pain, cough, and dyspnea related to the mass effect. The chest CT scan allows us to specify clearly the size and the location of the tumor and helps in surgical planning [15]. Because the clinical features and radiographic appearance are not specific, the diagnosis of intrapulmonary SFTs is difficult to obtain before the surgical biopsy and these tumors are commonly misdiagnosed as other diseases, such as thymic neoplasia, teratoma, neurogenic tumor, malignant pleural mesothelioma, or lung cancer [16]. The definitive diagnosis is made after histologic evaluation and the surgery has to be the best way to obtain simultaneous diagnosis and treatment especially that the fine-needle aspiration biopsy and biopsy by bronchoscopy are not reliable enough to serve as a guideline for therapeutic decisions [11]. The tumor resection must be wide and complete to avoid locoregional recurrence [17–19]. Pedicled tumors can be excised safely with VATS [16]. We performed a left pneumonectomy because of the tumor size and its extension to the upper lobe. Immunohistochemistry allows differentiating SFTs from other neoplasms. These tumors are CD34-, vimentin-, and bcl2-positive and they are negative for cytokeratin, desmins, alpha-smooth muscle actin, and S-100 protein [2, 16].

Prognosis of SFTs depends on morphologic and pathologic findings. Benign and pedicled tumors have the best prognosis than malignant and sessile tumors [2, 19, 20]. However, due to the rarity of intrapulmonary localized fibrous tumors of the lung, several studies will be needed to clarify their clinicopathologic behaviors [2, 18]. In our case, the length of follow-up is too short to comment about long-term outcomes.

## 4. Conclusion

A solitary fibrous tumor arising from the lung parenchyma is extremely rare. Wide resection is essential to cure patient and to avoid recurrence. A long-term follow-up is needed.
